# HPV testing in routine cervical screening: cross sectional data from the ARTISTIC trial

**DOI:** 10.1038/sj.bjc.6603210

**Published:** 2006-06-13

**Authors:** H C Kitchener, M Almonte, P Wheeler, M Desai, C Gilham, A Bailey, A Sargent, J Peto

**Affiliations:** 1 Division of Human Development, University of Manchester, Hathersage Road, Manchester M13 0JH, UK; 2 Non-Communicable Disease Epidemiology Unit, London School of Hygiene and Tropical Medicine, Keppel Street, London WC1E 7HT, UK; 3 Division of Human Development, University of Manchester, Hathersage Road, Manchester M13 0JH, UK; 4 Department of Cytology, Central Manchester and Manchester Children's University Hospitals NHS Trust, Oxford Road, Manchester M13 9WL, UK; 5 Cancer Research UK Epidemiology and Genetics Unit, Institute of Cancer Research, Sutton, Surrey SM2 5NG, UK; 6 Department of Virology, Central Manchester and Manchester Children's University Hospitals NHS Trust, Oxford Road, Manchester M13 9WL, UK; 7 Division of Human Development, University of Manchester, Hathersage Road, Manchester M13 0JH, UK; 8 Non-Communicable Disease Epidemiology Unit, London School of Hygiene and Tropical Medicine, Keppel Street, London WC1E 7HT, UK; 9 Cancer Research UK Epidemiology and Genetics Unit, Institute of Cancer Research, Sutton, Surrey SM2 5NG, UK

**Keywords:** cervical screening, HPV detection, typing, cytology

## Abstract

To evaluate the effectiveness of human papillomavirus (HPV) testing in primary cervical screening. This was a cross-sectional study from the recruitment phase of a prospective randomised trial. Women were screened for HPV in addition to routine cervical cytology testing. Greater Manchester, attendees at routine NHS Cervical Screening Programme. In all, 24 510 women aged 20–64 screened with liquid-based cytology (LBC) and HPV testing at entry. HPV testing in primary cervical screening. Type-specific HPV prevalence rates are presented in relation to age as well as cytological and histological findings at entry. In all, 24 510 women had adequate cytology and HPV results. Cytology results at entry were: 87% normal, 11% borderline or mild, 1.1% moderate and 0.6% severe dyskaryosis or worse. Prevalence of HPV decreased sharply with age, from 40% at age 20–24 to 12% at 35–39 and 7% or less above age 50. It increased with cytological grade, from 10% of normal cytology and 31% of borderline to 70% mild, 86% moderate, and 96% of severe dyskaryosis or worse. HPV 16 or HPV 18 accounted for 64% of infections in women with severe or worse cytology, and one or both were found in 61% of women with severe dyskaryosis but in only 2.2% of those with normal cytology. The majority of young women in Greater Manchester have been infected with a high-risk HPV by the age of 30. HPV testing is practicable as a primary routine screening test, but in women aged under 30 years, this would lead to a substantial increase in retesting and referral rates. HPV 16 and HPV 18 are more predictive of underlying disease, but other HPV types account for 30% of high-grade disease.

Cervical cancer is the second most common cancer among women worldwide, and ranks first in many developing countries (Parkin, 2004). Human papillomaviruses (HPV), most frequently HPV 16, are the primary cause of cervical carcinogenesis (IARC, 1995). Over 100 HPV types have now been described, including about 20 ‘high-risk’ types that are associated with cervical cancer (Clifford *et al*, 2005). The overall prevalence of HPV in cervical cancers in a large international study was over 99%, implying the highest attributable fraction ever identified for a specific cause of a major human cancer (Walboomers *et al*, 1999). In many developed countries, particularly the UK, systematic cervical screening based on cytology has been responsible for a significant fall in the incidence and death rate from cervical cancer (Peto *et al*, 2004). The NHS Cervical Screening Programme in England now offers screening 3 yearly between ages 25 and 49 and 5 yearly between 50 and 64 years, and liquid-based cytology (LBC) is currently being implemented. Nonrandomised studies have shown that HPV DNA testing is more sensitive than cytology for detecting CIN, and the International Agency for Research on Cancer (IARC) recently concluded that testing for HPV as a primary screening modality could reduce cancer incidence and mortality (IARC, 2004).

In order to evaluate the effectiveness of HPV testing in primary cervical screening, the Trial, A Randomised Trial in Screening to Improve Cytology (ARTISTIC) Trial is being conducted within the routine NHS Cervical Screening Programme in Greater Manchester. This randomised trial will compare outcomes in women whose HPV test result is concealed with those in whom it is revealed and acted upon. In this paper, we report prevalence rates of HPV 16, HPV 18 and other high-risk HPVs in relation to age, cytology and histology at entry to the trial.

## METHODS

Between July 2001 and October 2003, women aged 20–64 years attending for routine cervical screening in four health authorities in the Manchester area (Manchester, Salford & Trafford, Stockport and Wigan & Leigh) were invited to participate. The trial, which was approved by the North West Multicentre Research Ethics Committee, was explained in a letter enclosed with the invitation to attend for a routine smear. Women willing to participate were asked to sign informed consent before having a smear collected for LBC and HPV testing. This is a population-based study and is therefore representative of women in Greater Manchester, a heavily populated British conurbation.

The cervical sample was collected using the broom-like device of the Thin-Prep™ (Cytyc) kit and rinsed into a vial containing PreservCyt® transport medium. The sample and consent form were sent to the Manchester Cytology Centre laboratory in Manchester Royal Infirmary if they were collected in Manchester, Salford & Trafford, or Wigan & Leigh, and to Stepping Hill Hospital cytology laboratory if they were from the Stockport area. Samples accompanied by a consent form were flagged on receipt on the laboratory database. Consent forms were checked and forwarded to the ARTISTIC trial office, which sent a letter notifying participants of their random allocation (HPV result revealed or concealed).

### Design of the trial

**Figure 1:**
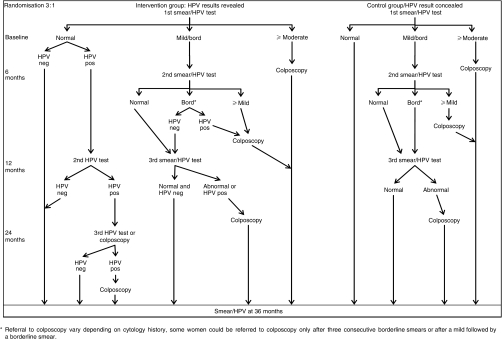
ARTISTIC Trial protocol for the management of women with normal and abnormal cytology and HPV-positive and -negative tests, in the revealed arm.

The data reported in this study are derived from the cytology and HPV testing performed during the recruitment phase. Figure 1 shows the design of the trial which will be completed in 2006/7. Women were randomised in a 3 : 1 ratio to the revealed arm or the concealed arm. In both arms the cervical sample was processed for cytology and then sent to the virology laboratory. HPV testing and cytology were thus conducted independently. Women with inadequate cytology at first visit were resampled. All women are being recalled for a second round of screening by LBC and HPV testing at 36 months.

### Concealed arm

All HPV results at entry and subsequently are concealed from the women and from clinical staff including the HPV results at 36 months. All women in the concealed arm, and those in the revealed arm who remained HPV-negative, were referred to colposcopy according to national guidelines at that time, that is (i) after three consecutive inadequate samples; (ii) after three consecutive borderline smears; (iii) after an initial borderline or mildly dyskaryotic smear followed by another showing mild dyskaryosis or worse; or (iv) following a single smear showing moderate dyskaryosis or worse.

### Revealed arm

Women in the revealed arm with normal cytology at entry who tested HPV positive were invited for a second HPV test 12 months after entry. If this second test was also positive, they were invited to choose between immediate colposcopy or a repeat HPV test after a further 12 months. If this third test was again positive, women were referred to colposcopy.

### Outcome measures

Primary outcome measures will include the proportion of additional CIN3 lesions diagnosed in the revealed arm in cytologically normal women who were HPV positive at entry and any resulting reduction in their CIN3 detection rate at the 3 year second screening round (CIN3 cases diagnosed in women who are cytologically normal at 3 years will be ignored in this concealed/revealed comparison). Other relevant outcomes are (a) the sensitivity and specificity of persistently detectable HPV for diagnosis of CIN3 or cancer; (b) the cost effectiveness of HPV testing; (c) the psychological effects of HPV testing; (d) attendance rates among HPV positive women when recalled after 1 year for retesting and (e) their preference between immediate colposcopy and further HPV testing if they are still HPV positive. Some HPV positive women fail to return for a second HPV test after 12 months, and the trial will also provide substantial evidence on the effects of retesting HPV positive women after 3 years, which may prove as sensitive but more specific for CIN3 diagnosis compared with retesting after 1 year.

### Laboratory procedures

#### LBC using ThinPrep®

ThinPrep® vials were collected from the participating practices or clinics and sent either to Manchester Cytology Centre or to Stepping Hill Hospital Cytology Laboratory. Vials not accompanied by a consent form were not processed until the consent form was received at the laboratory. Laboratory numbers were allocated to the samples before automatic staining and routine processing in cytology.

Most were processed using the multi sample ThinPrep® 3000 processor (Cytyc). The single sample ThinPrep® 2000 (Cytyc) was used if an extra slide needed to be made from a sample because the initial slide appeared unsatisfactory or if only a few samples needed to be processed.

Vials containing the residual sample were sent to the Virology Laboratory in Manchester Royal Infirmary for HPV testing by the Digene Hybrid Capture® 2 (hc2) assay. Laboratory numbers were allocated to the samples before automatic staining and routine processing. Procedures are in place to ensure that cross contamination by HPV does not occur in the T3000 processor and swabs are taken to ensure no contamination is present.

### HPV testing using Hybrid Capture® 2 (hc2)

#### Detection of high-risk HPV genotypes by hc2 assay

Cervical samples collected into Cytyc LBC medium were tested for high-risk HPV DNA by hc2 according to the manufacturer's instructions. Briefly, after treatment with conversion buffer, single-stranded DNA (ssDNA) was produced by adding denaturing fluid and heating at 65°C for 45 min. Following hybridisation of ssDNA to high-risk HPV specific RNA probes the DNA/RNA hybrids were captured onto anti-DNA/RNA antibody coated micro-titre plates prior to detection of the hybrids using an alkaline phosphatase conjugated anti-DNA/RNA antibody in conjunction with a light emitting substrate. Positive results were expressed in relative light units (RLUs) compared with a positive control containing 1 pg ml^−1^ of HPV DNA. The high-risk HPV types detected by the assay are 16, 18, 31, 33, 35, 39, 45, 51, 52, 56, 58, 59 and 68. We classified samples as HPV positive according to the manufacturer's instructions at the outset of the study, which was to use a positive cut off as 1 RLU/control.

### HPV genotyping (Roche reverse line blot assay)

Genotyping of hc2 positive samples was carried out using the prototype reverse line blot assay supplied by Roche.

DNA was extracted from 50 *μ*l of the pelleted sample stored at −70°C using the automated Roche MagNAPure LC system. Extracted DNA was amplified using the PGMY primer reagents provided by Roche. Biotinylated PCR product was then denatured and captured onto nylon strips coated with HPV type specific oligonucleotides. Immobilised product was visualised using streptavidin-horseradish peroxidase-mediated colour precipitation.

### Data management

Trial participants were flagged on the routine cytology and histology records of both cytology laboratories. In order to avoid the possibility that any histology results have been obtained from the second screening round, we have censored histology records at 2 years. Name, address and date of birth are routinely used by cytology laboratories to match new smears against a woman's previous screening record. A separate database was maintained by the virology laboratory. Relevant results were sent to the trial office in Manchester regularly, where they were matched and appended to participants' records on an Access 2000 database. Stata version 8 statistical software (StataCorp., 2004) was used for all analyses.

## RESULTS

A total of 25 020 women were enrolled of whom 397 (1.6%) had an inadequate first smear at entry including 71 (0.3%) who did not provide an adequate repeat smear. These 71 women were referred for colposcopy according to national guidelines and excluded from further analysis. The cytology results presented thus correspond to the first adequate smear. In all, 85 women had a conventional smear and no HPV test at entry, 137 had samples that were insufficient for HPV testing, 84 were younger than 20 and 133 were older than 64 years of age. There were thus 24 510 (18 386 revealed and 6124 concealed) eligible women with satisfactory cytology and HPV results at entry.

### Cytology and HPV results

**Figure 2:**
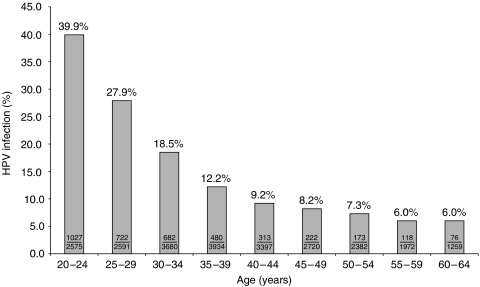
Prevalence of high-risk HPV (HR HPV) by Hybrid Capture 2 (HC2) according to age quinquennia.

Table 1 shows that cytology and HPV results at entry did not differ between the randomised arms and are therefore presented together; 87% of women had normal cytology, 11% had borderline smears or mild dyskaryosis, 1.1% had moderate dyskaryosis, and 0.8% had severe dyskaryosis or worse abnormality. The mean age was 40.2 years in both arms. The prevalence of high-risk HPV detection (Figure 2) declined from 40% in women aged 20–24 to 12% at 35–39 and 6% at 55 years or older.

**Figure 3:**
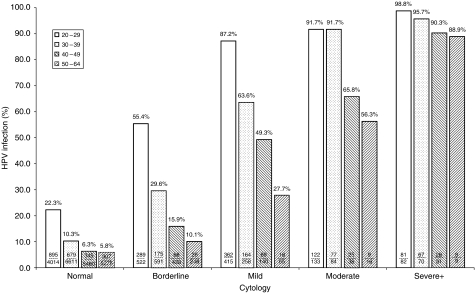
Prevalence of high-risk HPV (HR HPV) by Hybrid Capture 2 (HC2) by cytology grade within different age bands.

Figure 3 shows the prevalence of HPV infection by age and cytology at entry. HPV prevalence increased with the level of cytological abnormality, proportionally more so at older ages. HPV prevalence rates at age 20–29 years were 55% (borderline), 87% (mild), 92% (moderate) and 99% (severe). By age 50–64 years these rates had fallen to 10% (borderline), 28% (mild), 56% (moderate) and 89% (severe).

Table 2 shows the prevalence of abnormal cytology by age and HPV detection. The overall proportion of women with high-grade (moderate or severe) cytology declined from 4.2% (215/5166) at age 20–29 to 1.6% (223/13,731) at age 30–49. This marked reduction in high-grade disease appears to be due entirely to the much lower overall HPV prevalence in women aged 30–49 years, as the prevalence of high-grade abnormality in HPV-positive women was identical at age 20–29 years (11.6%: 203/1749) and at age 30–49 (11.6%: 197/1697). Above age 50, however, high-grade cytological abnormality was diagnosed in only 4.6% (17/367) of HPV-positive women and in 0.4% overall. The number of women who would have been referred for follow-up if primary screening with HPV testing replaced cytology can also be seen in Table 2. The proportion of smears that were abnormal was 22% (1153/5166) at aged 20–29 years, 12% (1640/13731) at age 30–49 years and 6.0% (338/5613) at age 50–64 years. The proportions HPV positive were 34% at 20–29 years, 12% at 30–49 years and 6.5% at 50–64 years.

Table 3 shows the proportions of women who tested positive for HPV 16, positive for HPV 18 (but not 16) and positive for hc2 in the absence of HPV 16 or HPV 18, in relation to age, cytology and histology at entry. The overall prevalence was 3.3% for HPV 16 and 1.1% for HPV 18. Among women with any high-risk type detected by hc2, the proportion who had HPV 16 was 31% at age 20–24 years, 23% at 25–34 years and 12% at 35–64 years. As the grade of cytological abnormality increased the proportion of women with HPV 16 infections increased from 1.5% in women with normal cytology to over 50% in those with severe dyskaryosis.

The lower part of Table 3 shows histological findings (worst histology within 2 years) among 1061 women with abnormal cytology at entry for whom a biopsy was obtained. No histology is yet available for the remaining 2069 women with an abnormal entry smear (bottom line, Table 3: abnormal cytology resolved or still being followed-up cytologically). Among women with histology, CIN3, CGIN or cancer was diagnosed in 46%, (150/325) of women with HPV 16, 28% (20/72) of those with HPV 18 (not 16), 20% (81/411) of those with other HPVs, and in only 3% (8/253) of those whose entry smears were hc2 negative. The hc2 test was positive in 92% of women with CIN2 and 97% with CIN3, CGIN or cancer. HPV 16 was detected in 59% of women with CIN3 or squamous cell carcinoma. HPV 18 was detected in 38% of women with CGIN or adenocarcinoma but in only 6% with CIN3 or squamous cell carcinoma.

## DISCUSSION

The ARTISTIC trial cohort represents the largest population of women in the UK to have undergone routine cervical screening with both LBC and HPV testing. The study population spanned the 20–64 age range of screened women when the trial opened, although the lower age threshold for routine cervical screening in England has since been increased from 20 to 25 years.

Our age-specific HPV positive rates in different grades of cytological abnormality were similar to those in the HART study in which over 10 000 women were screened with conventional cytology and hc2 testing, but our overall HPV prevalence was slightly higher at each age. HPV prevalence in the HART study declined from 14.5% in women aged 30–34 years to 3.8% in women aged 55–59 years (Cuzick *et al*, 2003); the corresponding rates in our cohort were 18.5% at 30–34 years and 6% at 55–59 years. Our higher rates may be partly due to regional differences in the UK (2004). The HART study was conducted in five centres across Britain, and the highest HPV prevalence was found in the Manchester area, where 16% of 30–34 year olds were positive for HPV (P Sasieni, personal communication). There may also have been a continuing increase in HPV prevalence in this population. A study conducted in the same area as ARTISTIC between 1988 and 1993 reported HPV prevalence based on MY0911 consensus primer PCR of 18% in women aged 20–24 years declining to 3% in women aged 50–54 years (Peto *et al*, 2004). Differences in HPV detection sensitivity may account for part of the disparity, but this doubling of prevalence within 12 years suggests a continuing epidemic rise in HPV prevalence.

Several useful conclusions relevant to the potential role for HPV testing in primary routine screening are suggested by the relationships between age, HPV detection and severity of cytology shown. In women with detectable HPV the prevalence of moderate dyskaryosis is 20- to 30-fold higher than in HPV negative women at all ages, and severe dyskaryosis is increased more than 100-fold. The prevalence of mild dyskaryosis in HPV-positive women is about 10-fold higher than in HPV negative women below age 50 years and more than five-fold higher above age 50 years. Although a great majority (87%) of women aged under 30 years with mild dyskaryosis are HPV positive, this proportion falls to 58% (233/398) at age 30–49 years and to only 28% (18/65) at age 50–64 years suggesting a role for HPV triage. The prevalence of borderline abnormalities in HPV positive women is about twice as high as in HPV-negative women at each age, and although there may be some overcalling by LBC, our results indicate that many borderline abnormalities are not caused by HPV. The prevalence of moderate or severe dyskaryosis in HPV-positive women is 11.6% throughout the premenopausal years suggesting that the natural history of HPV infection may be much the same in premenopausal women irrespective of age, although CIN3 is rarer in HPV-infected women aged 50 years or over. In women aged 30 years or over, our data suggest that the main effect of replacing cytology by HPV testing in primary screening would be the replacement of HPV negative abnormal smears, most of which would be borderline, by a similar number of HPV positive normal smears among women referred for follow-up. For those aged 20–29 years, however, the number who were HR HPV positive was 52% greater than the number with abnormal cytology, suggesting the need for a secondary test prior to colposcopy.

Primary screening with HPV testing in combination with cytology triage has been recommended only in women aged over 30 years (Sasieni and Cuzick, 2002; Cuzick *et al*, 2003), as HPV is so common in younger women. This conclusion seems questionable in the light of our results as high-grade dysplasia is as common among HPV-infected women aged under 30 years as in those age 30–49 years, and much commoner than in women aged over 50 years.

The ARTISTIC trial has also provided the largest collection of HPV typed primary screening cervical samples from the UK. The HPV type may be clinically important as the proportion of hc2 positive women who were infected with HPV16 increased with cytological abnormality, from 14% in those with normal cytology to 55% in those with severe dyskaryosis (Table 3). The HPV type might be used to determine whether to refer for colposcopy immediately, repeat the test, or defer any investigation until the next routine screen 3 years later. With the prospect of type 16/18 specific HPV prophylactic vaccines becoming available, data on these types in the screened population is of considerable importance in terms of what proportion of current abnormalities may still occur, notwithstanding a degree of cross protection reported for HPV 45 (Harper *et al*, 2006).

Women are still undergoing repeat cytology and colposcopy as necessary. It is therefore not yet possible to compare the sensitivity and specificity of cytology and HPV testing for detecting CIN2 and CIN3 at entry to the trial, particularly in relation to specific HPV types. As with any quantitative test, the performance of hc2 will depend on the choice of cutoff.

The second round of screening at 3 years began in July 2004 and we anticipate reporting final results at the end of 2007.

## Figures and Tables

**Table 1:** Cytology and high-risk HPV results by randomisation arms at entry

	**Revealed arm**			**Concealed arm**		
**Cytology**	**HPV −**	**HPV +**	**Total**	**HPV −**	**HPV +**	**Total**
Negative	14 367	1675	16 042 (87.3%)	4787	551	5338 (87.2%)
Borderline	923	420	1343 (7.3%)	309	137	446 (7.3%)
Mild	196	447	643 (3.5%)	69	166	235 (3.8%)
Moderate	34	170	204 (1.1%)	4	63	67 (1.1%)
Severe/worse	6	148	154 (0.8%)	2	36	38 (0.6%)
						
Total	15 526	2860	18 386 (100%)	5171	953	6124 (100%)

**Table 2:** Cytological abnormality by age and high-risk HPV detection

**Age**	**Normal**	**Borderline**	**Mild**	**Moderate**	**Severe**	**Total**
*20–29*						
HPV+	51.2% (895)	16.5% (289)	20.7% (362)	7.0% (122)	4.6% (81)	100% (1749)
HPV−	91.3% (3119)	6.8% (233)	1.5% (53)	0.32% (11)	0.03% (1)	100% (3417)
						
*30–49*						
HPV+	60.3% (1024)	14.3% (243)	13.7% (233)	6.0% (102)	5.6% (95)	100% (1697)
HPV−	92% (11 067)	6.4% (776)	1.4% (165)	0.17% (20)	0.05% (6)	100% (12 034)
						
*50–64*						
HPV+	83.6% (307)	6.8% (25)	4.9% (18)	2.4% (9)	2.2% (8)	100% (367)
HPV−	94.7% (4968)	4.3% (223)	0.9% (47)	0.13% (7)	0.02% (1)	100% (5246)
						
*20–64*						
HPV+	58.4% (2226)	14.6% (557)	16.1% (613)	6.1% (233)	4.8% (184)	100% (3813)
HPV−	92.5% (19 154)	5.9% (1232)	1.3% (265)	0.18% (38)	0.04% (8)	100% (20 697)

( ): number of women HPV positive (negative) in that category.

**Table 3:** Prevalence of HPV 16, HPV 18 and other high risk HPV types by age, cytology and histology at entry

**Age**	**HC-II negatives**	**HPV 16**	**HPV 18 (not HPV 16)**	**Other HC-II positives^a^**	**Total**
20–24	1548 (60.1%)	315 (12.2%)	80 (3.1%)	632 (24.6%)	2575 (100%)
25–34	4867 (77.6%)	320 (5.1%)	127 (2.0%)	957 (15.3%)	6271 (100%)
25–34	6538 (89.2%)	112 (1.5%)	43 (0.6%)	638 (8.7%)	7331 (100%)
45–54	4707 (92.2%)	35 (0.7%)	15 (0.3%)	345 (6.8%)	5102 (100%)
55–64	3037 (94.0%)	21 (0.7%)	7 (0.2%)	166 (5.1%)	3231 (100%)
					
*Cytology*					
Normal	19 154 (89.6%)	318 (1.5%)	143 (0.7%)	1765 (8.2%)	21 380 (100%)
Borderline	1232 (68.9%)	125 (7.0%)	54 (3.0%)	378 (21.1%)	1789 (100%)
Mild	265 (30.2%)	152 (17.3%)	40 (4.6%)	421 (47.9%)	878 (100%)
Moderate	38 (14.0%)	107 (39.5%)	18 (6.6%)	108 (39.9%)	271 (100%)
Severe or worse	8 (4.2%)	101 (52.6%)	17 (8.8%)	66 (34.4%)	192 (100%)
					
*Histology*					
CIN1 or less	318 (40.8%)	104 (13.3%)	48 (6.2%)	310 (39.7%)	780 (100%)
CIN2	15 (7.1%)	85 (40.1%)	15 (7.1%)	97 (45.7%)	212 (100%)
CIN3/SCC	7 (2.7%)	157 (60.4%)	14 (5.4%)	82 (31.5%)	260 (100%)
CGIN/ADCC	3 (16.7%)	6 (33.3%)	6 (33.3%)	3 (16.7%)	18 (100%)
					
Abnormal cytology, no histology^**^	1200 (64.5%)	133 (7.1%)	46 (2.5%)	481 (25.9%)	1860 (100%)
					
Total	20 697 (84.4%)	803 (3.3%)	272 (1.1%)	2738 (11.2%)	24 510 (100%)

SCC: squamous cell carcinoma.

CGIN: cervical glandular intraepithelial neoplasia.

ADCC: adenocarcinoma.

a Not HPV 16 or HPV 18.

^**^ Women with abnormal cytology at entry but no histology (abnormal cytology resolved or still being followed-up cytologically).

## Appendix

*ARTISTIC Trial Study Group*: HC Kitchener, Principal Investigator

P Wheeler, Trial Co-ordinator; *Epidemiology/Statistics*: J Peto, M Almonte, C Gilham, C Roberts, S Moss; *Cytopathology*: M Desai, J Mather; *Health Economics*: R Dowie, B Stoykova; *Virology Studies*: A Bailey, A Sargent, A Turner, G Corbitt (deceased).
